# Arab gum coating enhances the bioavailability and therapeutic potential of nanoselenium in cisplatin-induced testicular toxicity

**DOI:** 10.1038/s41598-026-55810-4

**Published:** 2026-06-10

**Authors:** Samar Abdelbaset, Mai Alaa El-Dein, Mahmoud M. Zakaria, Amira Awadalla, Amoura M. Abou-El-Naga

**Affiliations:** 1 Department of Zoology, Faculty of Science, Mansoura University, Mansoura, 35516 Egypt; 2 Urology and Nephrology Center, Faculty of Medicine, Mansoura University, Mansoura, 35516 Egypt; 3 Urology and Nephrology Center, Center of Excellence for Genome and Cancer Research Center, Mansoura University, Mansoura, 35516 Egypt; 4 Oncology Center, Molecular Genetics and Cancer Research Department, Mansoura University, Mansoura, 35516 Egypt

**Keywords:** Arab gum, Cisplatin, Male infertility, Oxidative stress, Selenium nanoparticles, Inflammation, Genotoxicity and testicular toxicity, Biochemistry, Biotechnology, Cancer, Cell biology

## Abstract

While Cisplatin remains a cornerstone of oncological intervention, its induction of systemic oxidative stress arranges a deleterious cascade that compromises male reproductive homeostasis. Selenium nanoparticles (SeNPs) may protect against oxidative stress and capping them with Arab gum (AG) could enhance their therapeutic efficacy. This study investigated the comparative gonadoprotective potential of Selenium Nanoparticles (SeNPs) and Arab gum (AG)-coated SeNPs (AG-SeNPs) against Cis-induced testicular dysfunction. Forty-five male Wistar rats were randomized into nine experimental groups: four baseline controls (Normal, AG, SeNPs, and AG- SeNPs) and five treatment groups receiving AG, SeNPs, AG-SeNPs, or a physical mixture of AG + SeNPs following Cis administration. Assessment parameters included the gonadosomatic index, biochemical markers of oxidative stress (CAT, GPX, MDA), and pro-inflammatory cytokines (TGF-beta, TNF-α and IL-6). Testicular integrity was further evaluated via histopathology, PCNA protein expression (cellular proliferation), and DNA fragmentation analysis using the Comet assay and apoptotic markers (Caspase-3 and Cytochrome C release). Administration of AG-SeNPs demonstrated superior gonadoprotective efficacy compared to uncoated SeNPs or physical mixtures. AG-SeNPs significantly restored redox homeostasis, via augmenting antioxidant enzyme activities (CAT, GPX) and reducing lipid peroxidation (MDA). Furthermore, AG-SeNPs significantly downregulated inflammatory signaling (TGF-beta, TNF-α and IL-6) and mitigated DNA damage. The treatment preserved genomic stability and inhibited the intrinsic apoptotic pathway by suppressing Cytochrome C release and Caspase-3 activation. Histological examination confirmed the restoration of seminiferous tubular architecture and enhanced cellular proliferation (PCNA expression). These findings suggest that AG-coating enhances the therapeutic index of SeNPs, likely due to improved bioavailability and synergistic antioxidant properties. AG-SeNPs represent a promising nanomedicine-based strategy for mitigating the gonadotoxic side effects of cisplatin-based chemotherapy.

## Introduction

Infertility is defined as a couple’s failure to conceive after twelve months of unprotected intercourse. This condition constitutes a significant public health concern globally^1^. Male factors significantly influence around 10–15% of couples worldwide, accounting for 20–30% of infertility cases and contributing to almost 50 percent of overall infertility cases. Male factor infertility is often diagnosed when semen analysis parameters get below the World Health Organization (WHO) standard levels, including disorders such as oligospermia (reduced sperm count), impaired sperm motility, and abnormal morphology^2^.

Male infertility is mostly attributed to impaired spermatogenesis, the intricate process of transforming spermatogonial stem cells into fully matured spermatozoa. This is a strictly regulated process that may be readily disturbed by several reasons, including genetic abnormalities, hormonal imbalances, and other environmental pollutants^3^. While varicocele, erectile dysfunction, and spermatic duct blockage contribute to male infertility, the etiology of diminished testicular function remains unexplained in over 60 percent of cases^4^.

Remarkably, sperm DNA fragmentation has emerged as a critical factor, often serving as the underlying cause of unexplained reproductive failure. Oxidative stress has emerged as a significant underlying aspect among several causes of male infertility. Elevated concentrations of reactive oxygen species (ROS) can trigger damage to DNA, lipids, and proteins in sperm, leading to diminished sperm motility, count, and viability^5,6^.

Antineoplastic medicines, particularly cisplatin (Cis), a commonly used chemotherapeutic medication, have been identified for significant male reproductive damage. Cis-induced testicular damage leads to elevated reactive oxygen species (ROS) generation, mitochondrial dysfunction, and the activation of apoptosis in germ cells, resulting in morphological and functional impairment of the testes, reduced testosterone levels, and compromised spermatogenesis^7^.

Antioxidant treatment has garnered considerable attention as a viable intervention to mitigate the detrimental effects of oxidative stress on male infertility and reproductive damage induced by chemotherapy. Selenium, an important trace element crucial for optimal testicular development and spermatogenesis, serves as a potent antioxidant. Selenium nanoparticles (SeNPs) have superior bioactivity and reduced toxicity compared to their bulk equivalents, hence enhancing their antioxidant, anti-inflammatory, and cytoprotective properties^8^.

Subsequent advancements in nanomedicine have led to the development of Arab Gum-coated selenium nanoparticles (AG-coated SeNPs). Arab Gum is a natural polysaccharide distinguished by its antioxidant and stabilizing properties, significantly influencing the bioavailability, stability, and targeted administration of SeNPs^9^.

The growing evidence indicates that nanoparticle-based therapies, in particular AG-coated SeNPs, are an innovative and effective method to alleviate Cis-induced infertility in men. This work aimed to evaluate the protective properties of AG-coated SeNPs against Cis-induced testicular damage in male rats.

## Materials and methods

### Chemicals

Arabic gum, CAS Number: 9000-01-5, Lot Number: 822140. Nano selenium and cisplatin, EIMC Pharmaceuticals Co., Cairo, Egypt CAS Number: 15663-27-1. All additional reagents were of superior analytical grade and obtained from Sigma-Aldrich Chemical Company Egypt.

### Preparation of selenium nanoparticles

SeNPs were synthesized by reducing selenite, following the methodology of Zhang et al.^10^, with minor modifications. In summary, 1 g of chitosan and 0.8 g of vitamin. C was entirely dissolved in 100 mL of 1% (w/w) acetic acid to produce a CHITOSAN/Vitamin C solution. Subsequently, 5 mL of an aqueous selenite solution containing 0.2 g of sodium selenite was incrementally added to the chitosan/Vitamin C solution and agitated rapidly at 500–600 rpm.

### Coating of selenium nanoparticles with Arab gum

Selenium nanoparticles are coated with Arabic gum by hydrothermal technique. Coating selenium nanoparticles with gum Arabic is a simple procedure that improves their stability and biocompatibility. Following the synthesis of selenium nanoparticles by the hydrothermal technique, they are disseminated in an aqueous solution and combined with a prepared Arabic gum solution. The mixture is thereafter agitated, often at ambient temperature or with gentle heating (50 °C), facilitating the formation of a protective coating of Arabic gum around the nanoparticles. The coated particles are subsequently recovered using centrifugation or filtering, filtered to eliminate surplus Arabic gum, and dehydrated, occasionally employing freeze-drying techniques. This coating technique produces selenium nanoparticles that exhibit superior colloidal stability, greater dispersibility in aqueous environments, and improved biocompatibility. The Arabic gum coating safeguards the nanoparticles from oxidation and enhances their application in targeted medication delivery systems. The coating’s characteristics may be improved by modifying elements such as Arabic gum concentration, reaction duration, and temperature during procedure^11^.

### Nanoparticles’ characterization

Multiple techniques were used to assess the physicochemical characteristics of selenium nanoparticles (SeNPs), Arab gum (AG), and AG-coated SeNPs. The zeta potential and particle size were determined at 25 °C using a Zetasizer Nano ZS 90 (Malvern, UK). Transmission electron microscopy (TEM) was used in order to observe morphology (JEOL 2100, 80 kV, Tokyo, Japan). The UV–visible spectra were measured on a Shimadzu UV-1800 spectrophotometer (Kyoto, Japan) and the fourier transform infrared (FT-IR) spectral on a Vertex 70 spectrometer (Bruker, Germany) at the range of 4000–400 cm^−1^. A D8 ADVANCE diffractometer (Bruker, Germany) was used to perform the X-ray diffraction (XRD) analysis.

### Gas chromatography–mass spectrometry (GC–MS) of Arab gum (AG)

The chemical composition of AG was identified by gas chromatography–mass spectrometry (GC–MS, Thermo Scientific Trace GC-TSQ, USA) with a TG–5MS column.

### Animals and ethical approval

Forty-five healthy adult male Wistar rats (≈180 g) were obtained from VACSERA (Giza, Egypt) and kept in standard conditions (22–25 °C, 12 h of light/dark cycle) with free access to food and water in the Medical Experimental Research Center (MERC), Faculty of Medicine, Mansoura University, Mansoura, Egypt. Rats were permitted a two-week acclimatization period prior to research. The protocol was approved by the Animal Ethics Committee of Mansoura University (Approval No. MU-ACUC SC. PhD.23.06.9) in accordance with relevant institutional, national, and international guidelines for the care and use of laboratory animals.

### Experimental design of animal groups

Forty-five male Wistar rats were randomly divided into nine groups/five rats in each. The sample size of n = 5 per group (total N = 45) was established to balance adequate statistical power with the ethical principle of animal reduction (the 3Rs). Because Cisplatin induces profound, unambiguous pathological alterations, a large effect size was anticipated. Based on Cohen’s principles for one-way ANOVA, assuming a large effect size (f ≥ 0.55) and a significance level of α = 0.05, a sample size of five animals per group is sufficient to achieve a statistical power (1-β) of at least 80% across the nine experimental groups. This conforms to standard practices in in vivo nanotoxicology and pharmacological efficacy studies.

All treatment sets were administered daily for 45 successive days, starting after cisplatin (Cis) single injection as in Table 1.

**Table 1:** Experimental groups.

Group	Treatment
Control	Saline (0.9%, orally)
AG	AG (7.5 mg/kg, orally)^12^
SeNPs	SeNPs (0.5 mg/kg, orally)^13^
AG-coated SeNPs	SeNPs (0.5 mg/kg coated with AG 7.5 mg/kg, orally)
Cis	Cis (5 mg/kg, single i.p. dose on Day 1)^14^ + Saline (0.9%, orally)
Cis + AG	Cis (5 mg/kg, single i.p. dose on Day 1) + AG (7.5 mg/kg, orally)
Cis + SeNPs	Cis (5 mg/kg, single i.p. dose on Day 1) + SeNPs (0.5 mg/kg, orally)
Cis + AG-coated SeNPs	Cis (5 mg/kg, single i.p. dose on Day 1) + SeNPs 0.5 mg/kg coated with AG 7.5 mg/kg (orally)
Cis + AG + SeNPs	Cis (5 mg/kg, single i.p. dose on Day 1) + AG (7.5 mg/kg, orally) + SeNPs (0.5 mg/kg orally, as a mixture not coated nanoparticles)

### Evaluation and analysis

Rats were euthanized after a 45-day treatment period using a mixture of xylazine (6 mg/kg) and ketamine (75 mg/kg) via intraperitoneal injection^15^. Blood and tissue specimens were subsequently collected for biochemical and histological analysis.

#### Gonado-Somatic Index

The testes, epididymides, and seminal vesicles were taken out and weighed immediately after the sacrifice, then the ratio of organ to body weight was estimated^16^.

#### Sperm analysis

The left Caudal epididymis was collected from all experimental groups, to assess the seminal parameters according to the manufacturer’s protocol. Briefly, the collected epididymises were dissected, placed in HTF (Easy Check, China), followed by incubation in a metal bath for 5 min at 37 °C. The total sperm count, motility, and morphology were examined manually by using a bright field microscope^17^.

#### Biochemical analysis

##### Oxidative stress and antioxidant biomarkers

Testicular Samples were minced and homogenized. The homogenates were then centrifuged at 10,000 × g, 15 min at 4 °C. The activity of Catalase (Cat. #: CAT, CA2517), Glutathione Peroxidase (GPx, Cat. #: MBS545286) and Malondialdehyde (MDA, Cat. #: MD2529) levels were determined in the supernatant using commercial colorimetric assay-kits (Bio-Diagnostics, Giza, Egypt).

##### Inflammatory biomarkers

Blood samples were taken from the heart under xylazine and ketamine anesthesia before sacrifice, serum was separated (3000 RPM, 15 min) and the Transforming Growth Factor Beta (TGF-β) and Interleukin-6 (IL-6) analyzed using ELISA Kits (Cambridge, MA, USA).

##### Histopathological investigations

The testes were fixed in Bouin’s solution, processed routinely, and sectioned (5 µm). Slides were stained with H&E for general histology and Masson’s Trichrome for collagen fibers, then examined microscopically (Olympus®). The processed tissue sections were examined and evaluated by an experienced pathologist. To eliminate observer bias and ensure full objectivity, the pathologist was blinded to the identity of the experimental groups during the microscopic analysis.

#### Immunohistochemistry

Sections encased in paraffin were deparaffinized, rehydrated, and underwent heat-induced antigen retrieval utilizing EDTA buffer (pH 8.0). Subsequent to the inhibition of non-specific binding, the slides were treated with a primary anti-Caspase-3 antibody (R&D Systems, MAB835), followed by an HRP-conjugated secondary antibody (Dako). Immunoreactivity was detected using 3,3'-diaminobenzidine (DAB), and the sections were counterstained with Mayer’s hematoxylin. Negative controls were established by replacing the main antibody with normal serum. An equivalent immunohistochemistry technique was executed utilizing an anti-proliferating cell nuclear antigen (PCNA) antibody (R&D Systems, MAB1389).

##### Computer-assisted digital image analysis

Slides were photographed with an Olympus E420 camera mounted on an Olympus® microscope (10 × and 40 ×). Caspase-3, PCNA immunoexpression, and collagen fibers were quantified using Image J software (USA).

#### Flow cytometry

Cytochrome C and TNF-α levels in testicular homogenates were measured with a BD Biosciences Flow Cytometry Assay Kit with CAS numbers (9007-43-6, 94948-59-1 respectively). Supernatants (10,000 × *g*, 15 min, 4 °C) were analyzed on a FACs Canto II (BD, USA) with FlowJo software.

#### Comet assay

Sperm DNA damage was assessed by alkaline Comet Assay^18^. Fifty cells per sample were analyzed using Comet Score software (Tritek, USA), with tail length and tail moment used as DNA damage indices.

#### Statistical analysis

Data are expressed as mean ± standard error (n = 5). One-way ANOVA and Tukey’s post-hoc test were conducted using GraphPad Prism (GraphPad 9.3.1 Software, USA). The significance level was established at *P* < 0.05. The percentage changes relative to controls were assessed where relevant.

## Results

### Characterization of selenium nanoparticles (SeNPs) and Arab gum-coated SeNPs (AG-coated SeNPs)

**Fig. 1:**
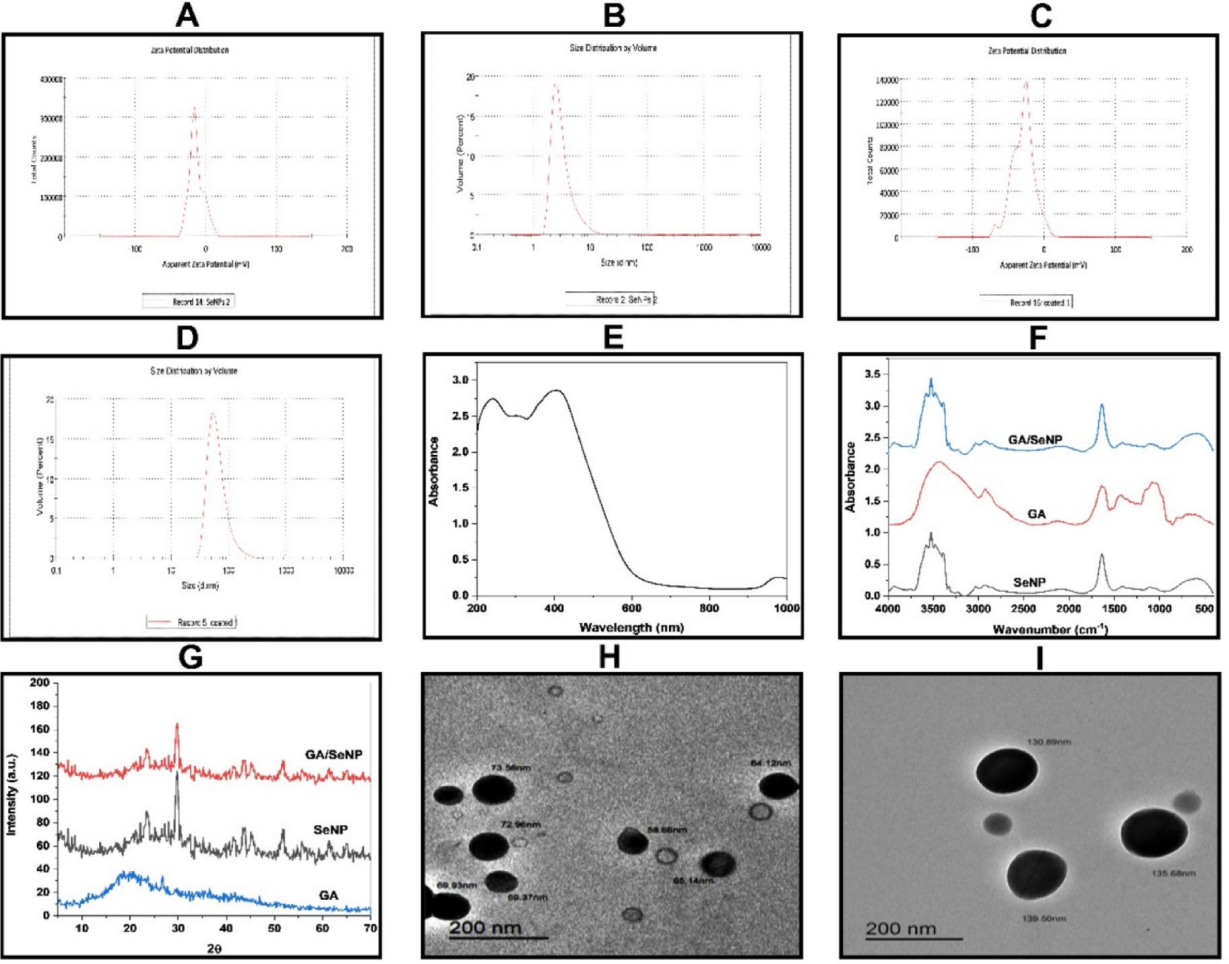
Characterization of selenium nanoparticles (SeNPs) and Arab gum-coated selenium nanoparticles (AG-coated SeNPs); (**A**): Zeta potential of SeNPs, (**B**): Particle size distribution analysis of SeNPs, (**C**): Zeta potential of AG-coated SeNPs, (**D**): Particle size distribution analysis of AG-coated SeNPs, **(E):** UV Visible spectroscopy analysis of SeNPs and AG-coated SeNPs, (**F**): Fourier transform infrared spectroscopy (FTIR) analysis of SeNPs and AG-coated SeNPs, (**G**): X-ray diffraction (XRD) analysis of SeNPs and AG-coated SeNPs, (**H**): Transmission electron microscope (TEM) characterization micrograph of SeNPs, (**I**): TEM characterization micrograph of AG-coated SeNPs.

Selenium nanoparticles (SeNPs) and Arab gum-coated selenium nanoparticles (AG-coated SeNPs) were evaluated by several analytical techniques. The zeta potential analysis revealed that the average zeta potential of SeNPs was − 13.7 mV (Fig. 1A), indicating moderate colloidal stability, whereas AG-coated SeNPs exhibited a significantly higher zeta potential of − 29.4 mV (Fig. 1C), hence demonstrating enhanced stability.

Dynamic Light Scattering (DLS) assessed the particle size distribution of SeNPs, revealing a unimodal distribution with a peak at 3.39 nm (Fig. 1B) and a polydispersity index (PDI) of 0.523. The DLS analysis of the AG-coated SeNPs indicated a peak size of 66.80 nm (Fig. 1D), with a Z-average value of 136.9 nm and a PDI of 0.453, indicating moderate heterogeneity.

UV–Visible spectroscopy demonstrated the effective application of the coating, as it displayed a characteristic absorption range of 250–300 nm for SeNPs (Fig. 1E) and a red shift for AG-coated SeNPs. The Fourier transform infrared (FTIR) and X-ray diffraction (XRD) techniques were employed to confirm the interaction between SeNPs and AG. FTIR revealed characteristic peaks in both samples (Fig. 1F), while XRD demonstrated sharp diffraction peaks for SeNPs and a broad amorphous signal for AG-coated SeNPs (Fig. 1G).

The TEM image of SeNPs revealed spherical particles with diameters ranging from 58.66 to 73.56 nm (Fig. 1H), whereas AG-coated SeNPs exhibited diameters between 130.89 and 139.50 nm (Fig. 1I), indicating effective coating. The particles exhibited a high degree of dispersion and little aggregation, indicating their suitability for biological applications.

The combined results of characterization of zeta potential, DLS, TEM, UV–Vis, FTIR and XRD analyses contain qualitative information of successful synthesis of SeNPs and its successful functionalization with AG. This close observation is a positive sign of the effective coating that resulted in an increased stability since AG coating significantly increased the colloidal stability of the nanoparticles which was evidenced by the presence of a high negative zeta potential. Besides it led to controlled morphology, in which the coated nanoparticles were similar in shape (sphere) and had increased size with a more robust and defined size.

It also resulted in preserved core structure since spectroscopy and diffraction study indicated that the interaction between the AG and SeNPs had been achieved and retained the crystalline structure of selenium core. These findings play a pivotal part in the production of stable and functional SeNPs to various possible biomedical and nanotechnological applications.

### Gas chromatography–mass spectrometry (GC–MS) analysis of AG and its potential impact on fertility

Table 2 presents a list of chemical constituents that have been detected by the GC/MS method along with their RT (Retention Time) and Composition (%). They are fatty acids, fatty acid derivatives and terpenes which are mostly of great importance in male infertility.

**Table 2:** List of AG active chemical constituents.

Entry	Chemical name	Classifacation	(RT, min.)	Molecular weight	Molecular formula	Composition%
1	1-(4-Isopropylphenyl)-2-methylpropyl acetate	Monoterpene	20.85	234	C_15_H_22_O_2_	2.27
2	Galaxolide	Sesquiterpene	24.67	258	C_18_H_26_O	1.99
3	Versalide	Sesquiterpene	25.00	258	C_18_H_26_O	2.11
4	Methyl hexadecanoate	Fatty acid derivative	26.56	270	C_17_H_35_O_2_	7.65
5	Hexadecanoic acid	Fatty acid	27.50	256	C_16_H_32_O_2_	9.53
6	Oleic acid	Unsaturated Fatty acid	27.59	282	C_18_H_34_O_2_	4.61
7	Linolelaidic acid, methyl ester	Fatty acid derivative	29.58	294	C_19_H_34_O_2_	8.63
8	Oleic acid, methyl ester	Fatty acid derivative	29.75	296	C_19_H_36_O_2_	20.96
9	Methyl stearate	Fatty acid derivative	30.29	298	C_19_H_38_O_2_	1.19
10	Linoleic acid	Unsaturated Fatty acid	30.58	280	C_18_H_32_O_2_	16.68
11	Stearic acid	Fatty acid	31.06	284	C_18_H_36_O_2_	1.68
12	Methyl gadoleate	Fatty acid derivative	32.04	324	C_21_H_40_O_2_	0.91
13	Erucic acid	Fatty acid	33.48	338	C_22_H_42_O_2_	3.48
14	13-Docosenoic acid, methyl ester	Fatty acid derivative	36.47	352	C_23_H_44_O_2_	12.69
	Total					94.38

This table gives a summary of the main organic compounds found in AG by GC–MS, including a special emphasis on individual compounds and the percentage cumulative areas where several entries of the same compound or close isomers are observed. Interestingly, the elevated composition percentages, particularly in compounds like Oleic acid methyl ester (20.96%) and Linoleic acid (16.68%), suggest their significant role in sustaining male fertility. The aforementioned compounds are recognized for maintaining the structural integrity of sperm membranes, enhancing motility, and regulating hormonal processes, all of which are essential for male reproductive health.

### Effect of AG, SeNPs and AG-coated SeNPs on the body weight and Gonado-Somatic index

**Fig. 2:**
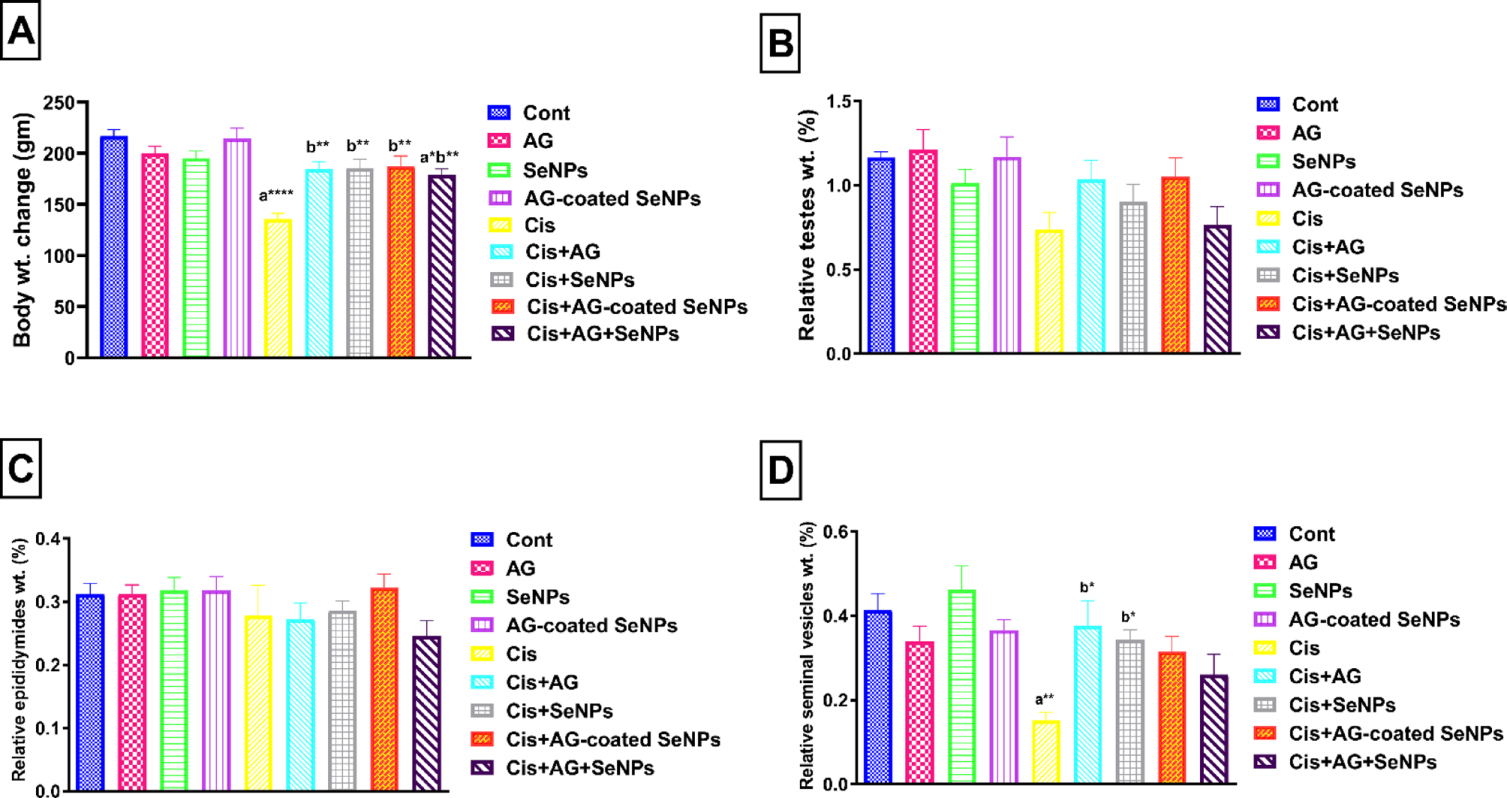
Impact of AG, SeNPs, and AG-coated SeNPs on Body Weight and Gonado-Somatic Index, (**A**): Body weight change (g), (**B**): Relative testes weight (%), (**C**): Relative epididymides weight (%), and (**D**): Relative seminal vesicles weight (%). Data are presented as mean ± SE (n = 5 per group). ‘a’ denotes significance vs. control; ‘b’ denotes significance vs. Cis, ‘c’ denotes significance Cis + AG, ‘d’ denotes significance vs. Cis + SeNPs. ‘e’ denotes significance vs. Cis + AG + SeNPs, respectively. *P < 0.05, **P < 0.01, and ****P < 0.0001.

Figure 2A–D illustrates that body weight (Fig. 2A) and relative seminal vesicles weight (Fig. 2D) were significantly decreased after Cisplatin (Cis) treatment (*P* < 0.0001 and *P* < 0.01, respectively) in correlation with the control group, which is evidence of systemic toxicity, but there was no significant effect on relative testes (Fig. 2B) and epididymides (Fig. 2C) weights.

In relation to Cis, body weight was significantly (*P* < 0.01) enhanced through co-treatments of AG, SeNPs, AG-coated SeNPs and AG + SeNPs, implying protection. While relative seminal vesicles weight only exhibit significant increase (*P* < 0.05) through co-treatments of AG, and SeNPs. Even though the relative testes and epididymides weights partially recovered, the changes were not significant. Overall, co-treatments, especially AG-coated SeNPs, demonstrated effective protection of Cis-induced damage, which represents a promising sign for preventing Cis-toxicity.

### Ameliorative effects of AG, SeNPs and AG-coated SeNPs on sperm quality

**Fig. 3:**
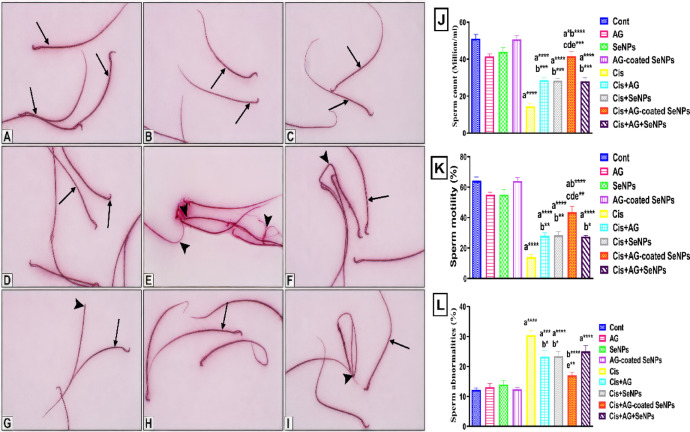
Impact of AG, SeNPs, and AG-coated SeNPs on sperm quality. (**A**)–(**D**) (Control, AG, SeNPs, and AG-coated SeNPs groups, respectively) all exhibit normal morphology characterized by a distinct hook-shaped head and a long, straight tail (arrows); (**E**) Cis group Displays a cluster of malformed sperm with amorphous heads and coiled or biforked tails (arrowhead); (**F**), (**G**), and (**I**) (Cis + AG, Cis + SeNPs, and Cis + AG + SeNPs groups, respectively) mild sperm abnormities (black arrowhead), whereas, (**H**) (Cis + AG-coated SeNPs) showing normal sperm morphology (black arrow) as the control group; (**J**) Sperm count (Million/ml), (**K**) Sperm motility (%), and (**L**) Sperm abnormalities (%). Data are presented as mean ± SE (n = 5 per group). ‘a’ denotes significance vs. control; ‘b’ denotes significance vs. Cis, ‘c’ denotes significance vs. Cis + AG, ‘d’ denotes significance vs. Cis + SeNPs. ‘e’ denotes significance vs. Cis + AG + SeNPs, respectively. * (*P* < 0.05), ** (*P* < 0.01), ), *** (*P* < 0.001), and **** (*P* < 0.0001).

The induction of gonadotoxicity by Cisplatin (Cis) resulted in a profound decline in sperm count (Fig. 3J) and motility (Fig. 3K), accompanied by a sharp increase in morphological abnormalities (Fig. 3L) (*P* < 0.0001 vs. Control). These alterations signify a disruption in the seminiferous tubular microenvironment and a failure of the spermatogenic process. Notably, while monotherapy with AG or SeNPs provided partial recovery, the AG-coated SeNP (Cis + AG-coated SeNPs) group demonstrated the most robust protective effect. This group showed a statistically superior restoration of sperm count and motility compared to both the uncoated SeNP group and the physical mixture (Cis + AG + SeNPs), effectively bringing these parameters toward baseline levels. Furthermore, the significant reduction in sperm abnormalities in the AG-coated SeNP group suggests a preservation of genomic and structural integrity during spermiogenesis. Morphologically, sperms in the Cis group depicted loss of the characteristic hook-shaped appearance typical of rrat sperm. The heads appear rounded, or misshapen. Complete detachment of the sperm head, leaving only the tail (flagellum) visible, which is a common indicator of structural fragility or oxidative damage during spermiogenesis. While the tail is tightly wrapped in a spiral or circular configuration. Treatment sets showed a marked enhancement in the sperm morphology, with the most beneficial improvement in the AG-coated SeNP group (Fig. 3 A–I).

### Impact of AG, SeNPs and AG-coated SeNPs on oxidative and antioxidant levels

**Fig. 4:**
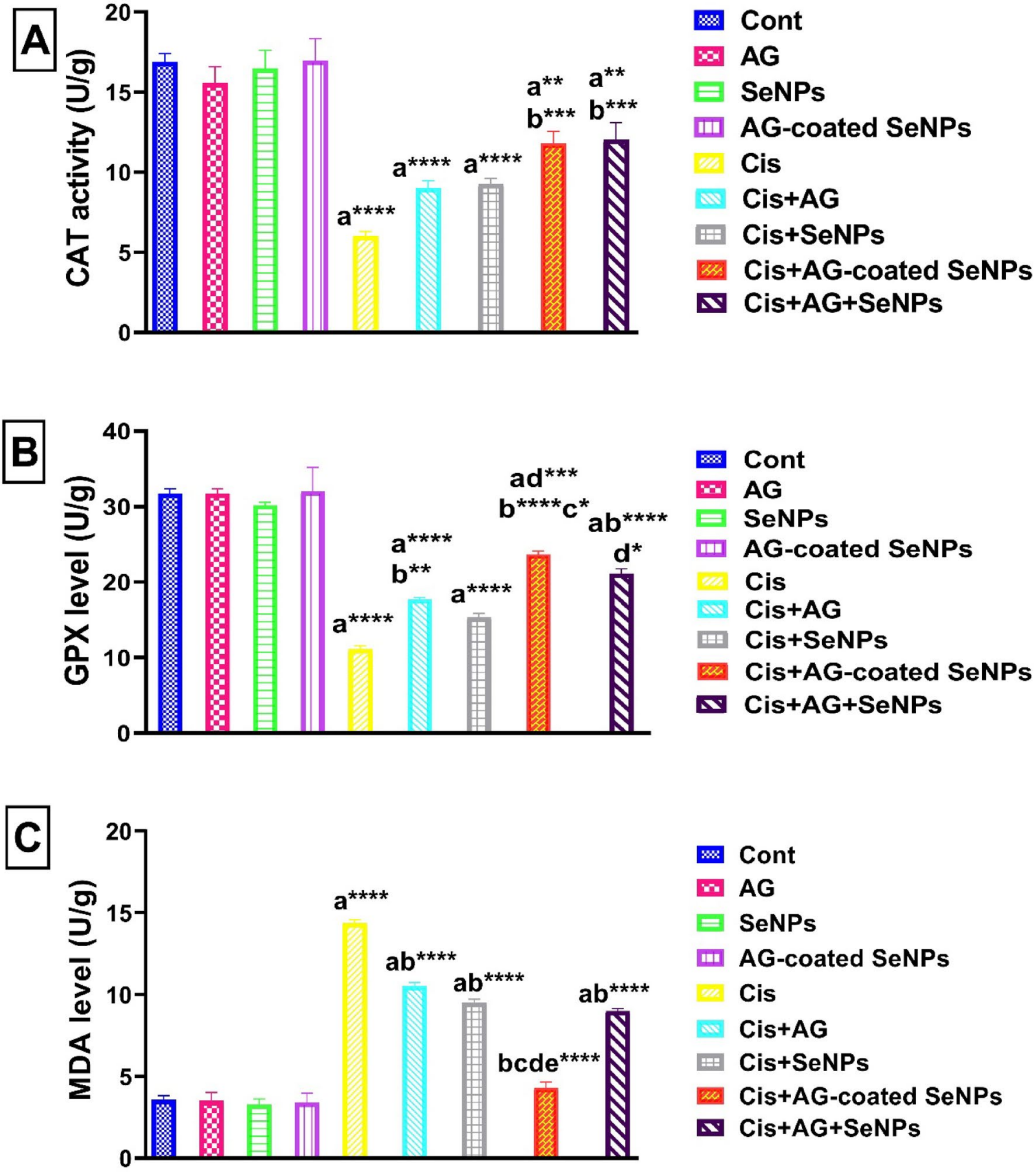
Impact of AG, SeNPs, and AG-coated SeNPs on Oxidative and Antioxidant Levels, (**A**) Catalase (CAT) activity, (**B**) Glutathione Peroxidase (GPX) levels, and (**C**) Malondialdehyde (MDA) levels in testicular tissue. Data are presented as mean ± SE (n = 5 per group). ‘a’ denotes significance vs. control; ‘b’ denotes significance vs. Cis, ‘c’ denotes significance vs. Cis + AG, ‘d’ denotes significance vs. Cis + SeNPs. ‘e’ denotes significance vs. Cis + AG + SeNPs, respectively. * (*P* < 0.05), ** (*P* < 0.01), ), *** (*P* < 0.001), and **** (*P* < 0.0001).

Figure 4A–C illustrates the effects of various treatments on Catalase (CAT) activity (Fig. 4A), Glutathione Peroxidase (GPX) (Fig. 4B), and Malondialdehyde (MDA) levels (Fig. 4C). The administration of Cis markedly (*P* < 0.0001) reduced both CAT activity and GPX levels, while increasing MDA levels relative to the control group, indicating the generation of oxidative stress. In contrast, AG-coated SeNPs and AG + SeNPs co-treatments demonstrated a substantial recovery of CAT activity (*P* < 0.001) and GPX levels (*P* < 0.0001), while AG, SeNPs, AG-coated SeNPs, and AG + SeNPs co-treatments significantly reduced MDA levels (*P* < 0.0001) compared to the Cis group. This demonstrates their antioxidant capacity. AG-coated SeNPs had the most significant protective effects across all treatment groups.

### Impact of AG, SeNPs, and AG-coated SeNPs on fibrotic and inflammatory cytokines

**Fig. 5:**
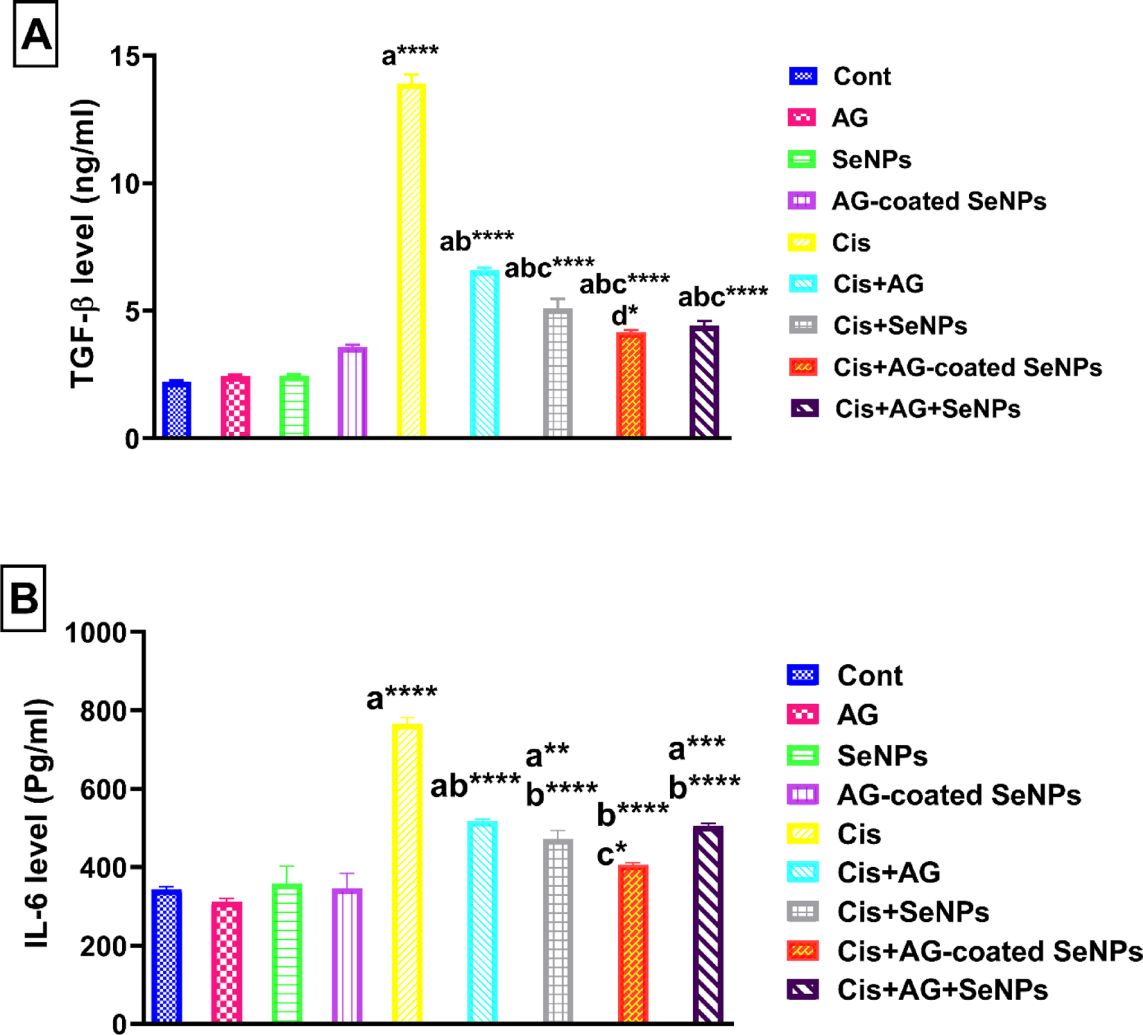
Impact of AG, SeNPs, and AG-coated SeNPs on serum Fibrotic and Inflammatory Cytokines. (**A**) TGF-β levels (ng/ml) and (**B**) IL-6 levels (pg/ml). Data are presented as mean ± SE (n = 5 per group). ‘a’ denotes significance vs. control; ‘b’ denotes significance vs. Cis, ‘c’ denotes significance vs. Cis + AG, ‘d’ denotes significance vs. Cis + SeNPs. ‘e’ denotes significance vs. Cis + AG + SeNPs, respectively. * (*P* < 0.05), ** (*P* < 0.01), ), *** (*P* < 0.001), and **** (*P* < 0.0001).

**Fig. 6:**
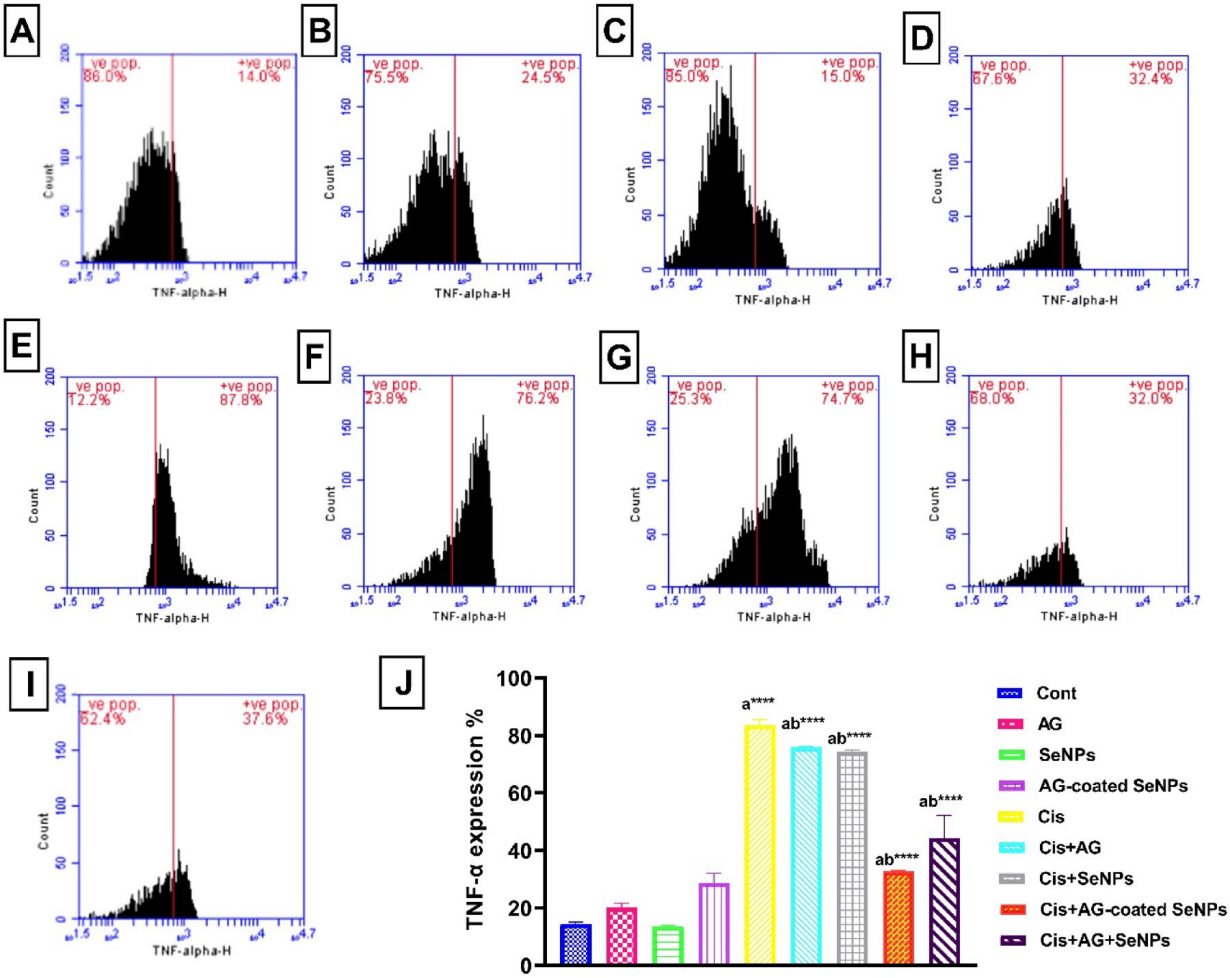
Impact of AG, SeNPs, and AG-coated SeNPs on testicular TNF-α level. (**A**) Control group, (**B**) AG group, (**C**) SeNPs group, and (**D**) AG-coated SeNPs group, (**E**) Cis group, (**F**) Cis + AG group, (**G**) Cis + SeNPs group, (**H**) Cis + AG-coated SeNP group, (**I**) Cis + AG + SeNPs group and (**J**) is the TNF-α expression %. ‘a’ denotes significance vs. control; ‘b’ denotes significance vs. Cis. * (*P* < 0.05), ** (*P* < 0.01), ), *** (*P* < 0.001), and **** (*P* < 0.0001).

Figure 5 illustrates the impact of various therapies on cytokine levels and offers significant insights into fibrotic and inflammatory responses. Figure 5A and B demonstrates that serum Transforming Growth Factor Beta (TGF-β) and Interleukin-6 (IL-6), respectively, substantially elevated in the Cis group relative to the control group (*P* < 0.0001). This suggests that Cis promotes fibrosis, immunosuppression, and inflammation. AG, SeNPs, AG-coated SeNPs, and AG + SeNPs co-treatments significantly lowered the elevated TGF-β and IL-6 levels (*P* < 0.0001) compared to the Cis group, indicating their protective capabilities against fibrosis and potent anti-inflammatory properties. On the whole, our findings suggest that Cis-induced fibrosis and inflammation may be effectively alleviated when Cis is combined with AG, SeNPs, or AG-coated SeNPs, with the Cis + AG-coated SeNPs combination being most promising. Flow cytometric analysis revealed that Cisplatin (Cis) administration triggered a dramatic elevation in Tumor Necrosis Factor-alpha (TNF-α) levels (*P* < 0.0001), confirming the induction of a robust pro-inflammatory state in the testicular tissue. While treatment with AG or SeNPs individually showed a marginal reduction in this cytokine, the Cis + AG-coated SeNP group exhibited the most significant attenuation of TNF-α. This nano-formulation outperformed both the uncoated SeNPs and the physical mixture (Cis + AG + SeNPs), effectively suppressing the inflammatory surge and restoring TNF-α expression toward baseline control levels (Fig. 6A–J).

### Histopathological findings

#### H&E staining

**Fig. 7:**
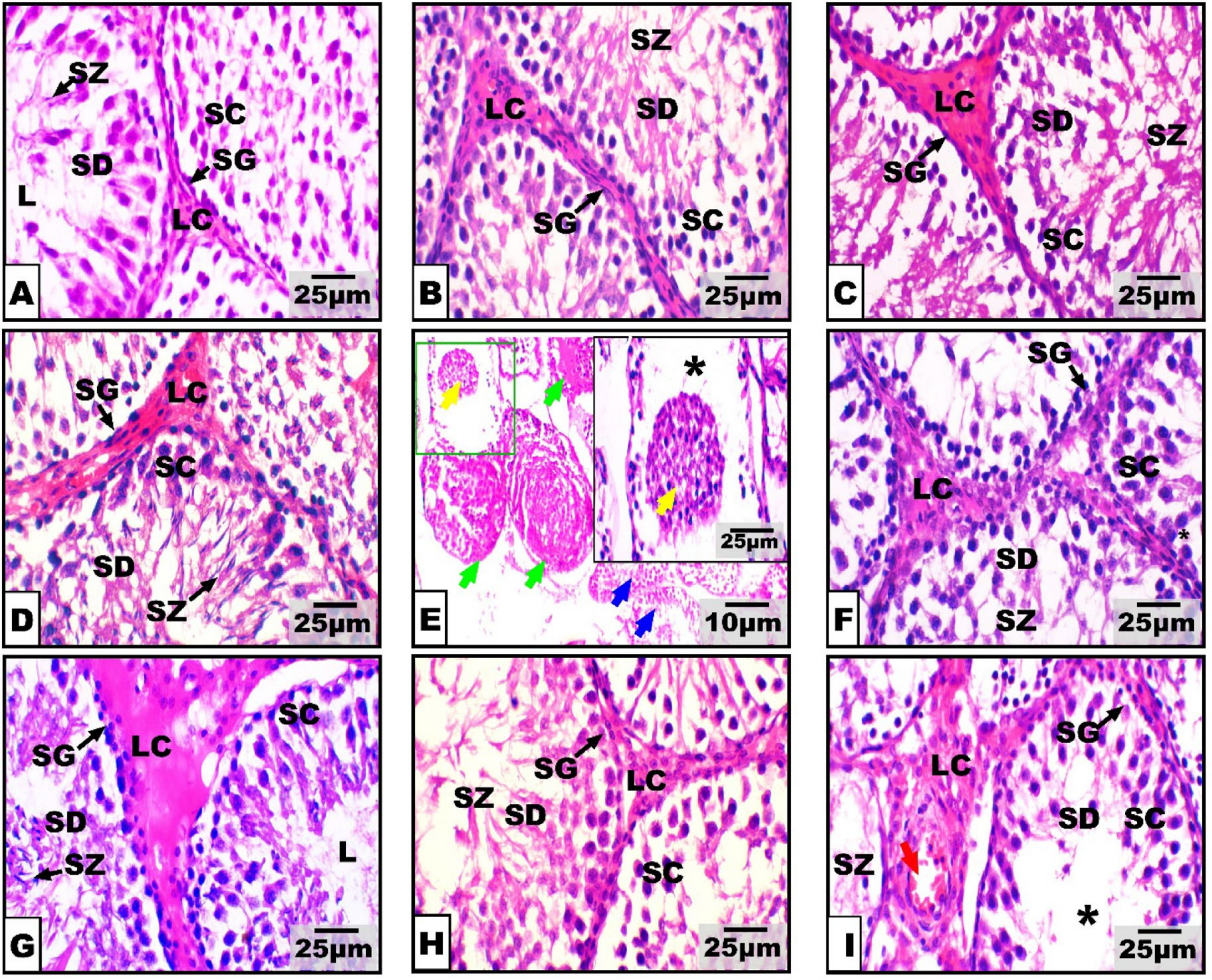
(**A**–**I**) Seminiferous tubule histoarchitecture across control and other experimental groups (Scale bar A-I = 25 µm, inset = 10 µm). (**A**)–(**D**) (Control, AG, SeNPs, and AG-coated SeNPs groups, respectively) all exhibit normal seminiferous tubule features. These include Normal spermiogenesis: Spermatogonia (SG), spermatocytes (SC), spermatids (SD), and spermatozoa (SZ). Normal lumen (L) and Leydig cells (LC) in the interstitial spaces. (**E**) (Cis group) demonstrates marked testicular disorganized seminiferous epithelium (blue arrows) accompanied with collapsed necrotic tubules (green arrows), a noticeable number of germinal cells desquamating and folding into the tubular lumen (yellow arrow) leaving wide intratubular spaces (asterisks). (**F**)–(**H**) (Cis + AG, Cis + SeNPs, and Cis + AG-coated SeNPs groups, respectively) show considerable restoration of seminiferous tubules: Lining by nearly normal layers of germ cells. Basement membranes of nearly normal thickness. **I** (Cis + AG + SeNPs group) indicates mild degeneration, congested blood vessel (red arrow). low density of spermiogenesis (asterisks).

Figure 7 illustrates the histoarchitecture of seminiferous tubules across the different experimental groups, as revealed by H&E staining. All control groups (Control, AG, SeNPs, and AG-coated SeNPs) displayed a typical seminiferous tubule architecture with an intact germinal epithelial layer (Fig. 7A–D).

Conversely, the Cis group (Fig. 7E) exhibited considerable damage to the testicles. This was marked by damaged seminiferous tubules, severe vacuolation, and reduced germ cell density, indicating of substantial cytotoxic damage. Tissue recovery was seen at different levels in the co-treatment groups (Cis + AG, Cis + SeNPs, Cis + AG-coated SeNPs, and Cis + AG + SeNPs) (Fig. 7F–I). Nonetheless, the Cis + AG-coated SeNPs group (Fig. 7H) exhibited enhanced structural recovery. This group exhibited improved seminiferous tubule organization, significantly reduced vacuolation, and a more intact germinal epithelium.

#### Masson’s trichrome staining

**Fig. 8:**
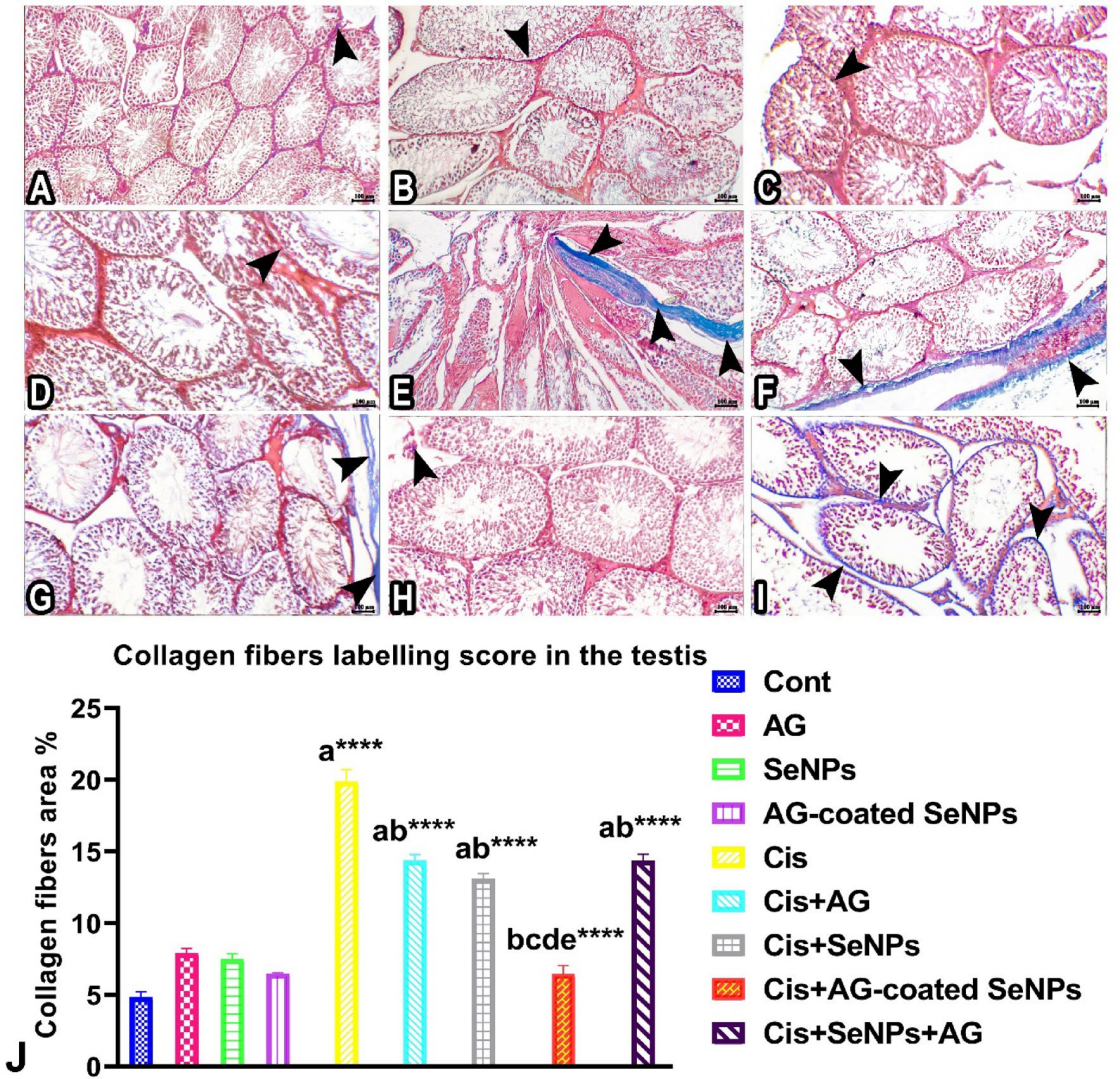
(**A**)–(**J**) Masson’s Trichrome-stained sections of testicular tissue from control and other experimental groups. (Scale bar = 100 µm).(**A**) Control group, (**B**) AG group, (**C**) SeNPs group, and (**D**) AG-coated SeNPs group showing negligible collagen deposition. (**E**) Cis group showing marked increase in collagen fibers. (**F**) Cis + AG group, and (**G**) Cis + SeNPs group showing mild reduction in collagen deposition compared to the Cis group. (**H**) Cis + AG-coated SeNPs group showing pronounced reduction in collagen fibers, closely resembling the control pattern. (**I**) Cis + AG + SeNPs group depicting a slight reduction in collagen fibers. Positive collagen fibers are indicated by black arrowheads. (**J**) Quantitative image analysis of collagen fibers area (%) in the testes of all groups. Data are expressed as mean ± SE (n = 5 per group). ‘a’ denotes significance vs. control; ‘b’ denotes significance vs. Cis, ‘c’ denotes significance vs. Cis + AG, ‘d’ denotes significance vs. Cis + SeNPs. ‘e’ denotes significance vs. Cis + AG + SeNPs, respectively. **** (*P* < 0.0001).

Table 3 and Fig. 8 demonstrated Masson’s Trichrome staining to assess collagen fiber deposition in the testes. Control groups; Fig. 8A–D (Control, AG, SeNPs, AG-coated SeNPs, respectively) exhibited insignificant presence of collagen fibers around the seminiferous tubules. Cis group; Fig. 8E, had a strong incidence of blue-stained collagen fibers in the testicular capsules. Co-treatment groups, (Cis + AG, Cis + SeNPs, and Cis + AG + SeNPs) Fig. 8 F, G and I, respectively, exhibited a significant decrease of these fibers, while the Cis + AG-coated SeNPs group, had the strongest anti-fibrotic effect Fig. 8H. The area of collagen fibers (%) in the testicular tissue of each group was quantified and provided in Fig. 8J.

**Table 3:** Collagen fiber distribution in the seminiferous tubules and testicular capsule (Masson’s Trichrome staining).

Group	Collagen fibers around seminiferous tubules	Collagen fibers in the testicular capsule	Overall change versus control
Control (Fig. 8A)	Negligible	Negligible	–
AG (Fig. 8B)	Negligible	Negligible	No change
SeNPs (Fig. 8C)	Negligible	Negligible	No change
AG-coated SeNPs (Fig. 8D)	Negligible	Negligible	No change
Cis (Fig. 8E)	Markedly increased (blue staining)	Markedly increased (blue staining)	Significant increase
Cis + AG (Fig. 8F)	Mild reduction compared to Cis	Mild reduction compared to Cis	Partial improvement
Cis + SeNPs (Fig. 8G)	Mild reduction compared to Cis	Mild reduction compared to Cis	Partial improvement
Cis + AG-coated SeNPs (Fig. 8H)	Pronounced reduction	Pronounced reduction	Significant improvement
Cis + AG + SeNPs (Fig. 8I)	Mild reduction compared to Cis	Mild reduction compared to Cis	Partial improvement

### Immunohistochemical investigations of Caspase-3 and PCNA in the testes

**Fig. 9:**
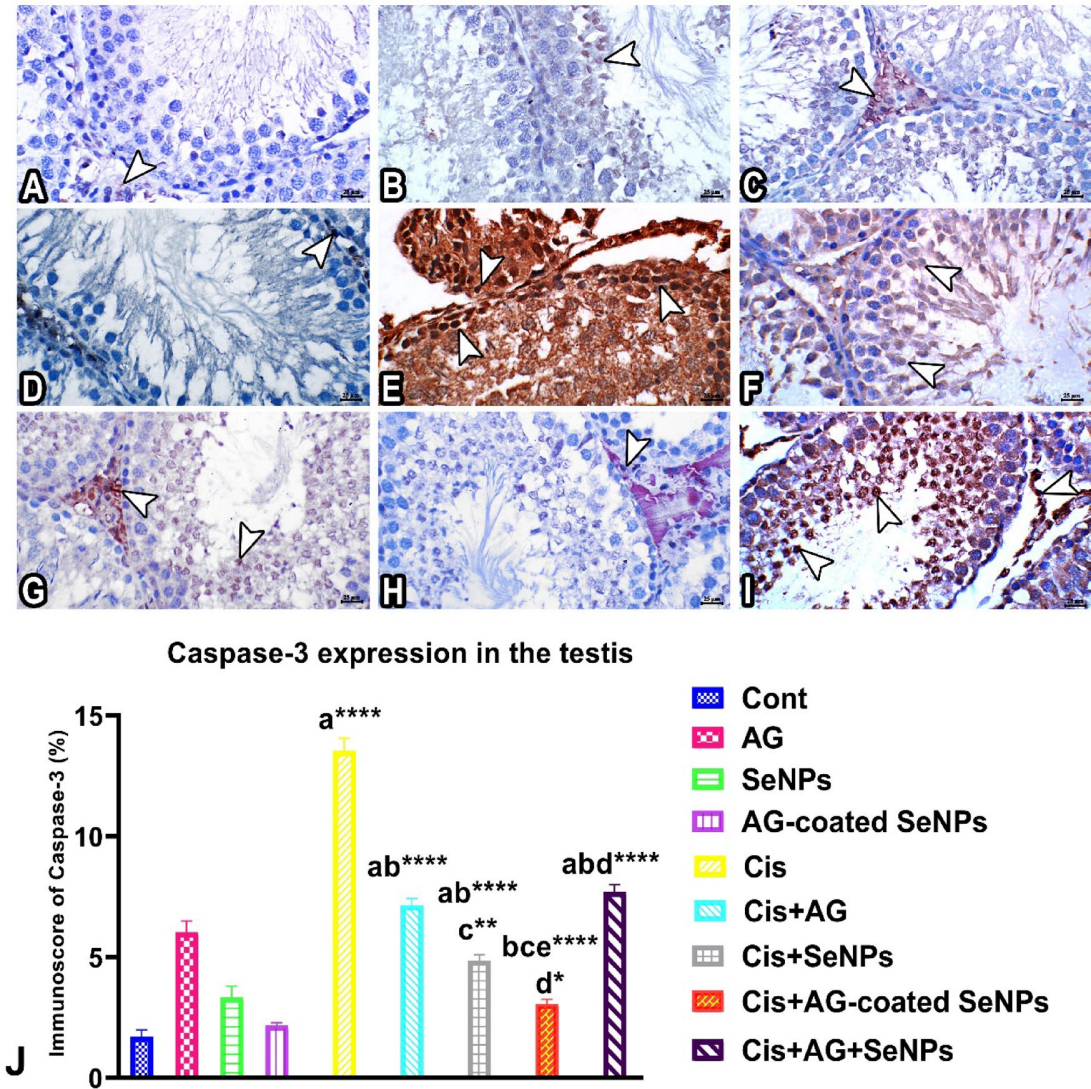
**A**–**J** Photomicrographs of Caspase-3 immunostained testicular tissue (Scale bar = 25 µm). (**A**) Control group, (**B**) AG group, (**C**) SeNPs group, and (**D**) AG-coated SeNPs group showing normal, mild Caspase-3 expression. (**E**) Cis group showing intense Caspase-3 expression. (**F**) Cis + AG group and (**G**) Cis + SeNPs group showing mild Caspase-3 expression. (**H**) Cis + AG-coated SeNPs group showing the lowest Caspase-3 expression through the treatment sets. (**I**) Cis + AG + SeNPs group showing mild Caspase-3 expression. Positive Caspase-3 immunoreactivity is indicated by white arrowheads. (**J**) Quantitative image analysis of Caspase-3 immunoreactivity (%) in the testes of all groups. Data are expressed as mean ± SE (n = 5 per group). 'a' denotes significance vs. control; 'b' denotes significance vs. Cis, 'c' denotes significance vs. Cis + AG, 'd' denotes significance vs. Cis + SeNPs. 'e' denotes significance vs. Cis + AG + SeNPs, respectively. * (*P* < 0.05), ** (*P* < 0.01) and **** (*P* < 0.0001).

Figure 9 displays the immunohistochemical labeling of Caspase-3 with the corresponding image analysis of Caspase-3 immunoreactivity (%) for all experimental groups. The control (Fig. 9A), AG (Fig. 9B), SeNPs (Fig. 9C), and AG-coated SeNPs (Fig. 9D) groups exhibited low Caspase-3 expression, indicating a baseline level of apoptotic activity. In contrast, the Cis group (Fig. 9E) exhibited a statistically significant increase (*P* < 0.0001) in Caspase-3 expression compared to the control group, strongly indicating apoptosis.

Conversely, co-administrations with AG (Cis + AG; Fig. 9F), SeNPs (Cis + SeNPs; Fig. 9G), AG-coated SeNPs (Cis + AG-coated SeNPs; Fig. 9H), and AG + SeNPs (Cis + AG + SeNPs; Fig. 9I) significantly decreased Caspase-3 expression relative to the Cis group (P < 0.0001), indicating a protective effect. The Cis + AG-coated SeNPs group exhibited the most significant reduction in Caspase-3 expression, indicating superior cytoprotective efficacy. The Cis + AG + SeNPs group exhibited higher Caspase-3 expression than the Cis + AG-coated SeNPs, but lower than the Cis group expression (*P* < 0.0001).

The quantitative analytical findings of Caspase-3 immunoreactivity, represented as a percentage across all groups, are shown in Fig. 9J. The data indicated that co-treatments with AG, SeNPs, and AG-coated SeNPs reduce Cis-induced testicular apoptosis, with AG-coated SeNPs demonstrating the most significant anti-apoptotic impact.

**Fig. 10:**
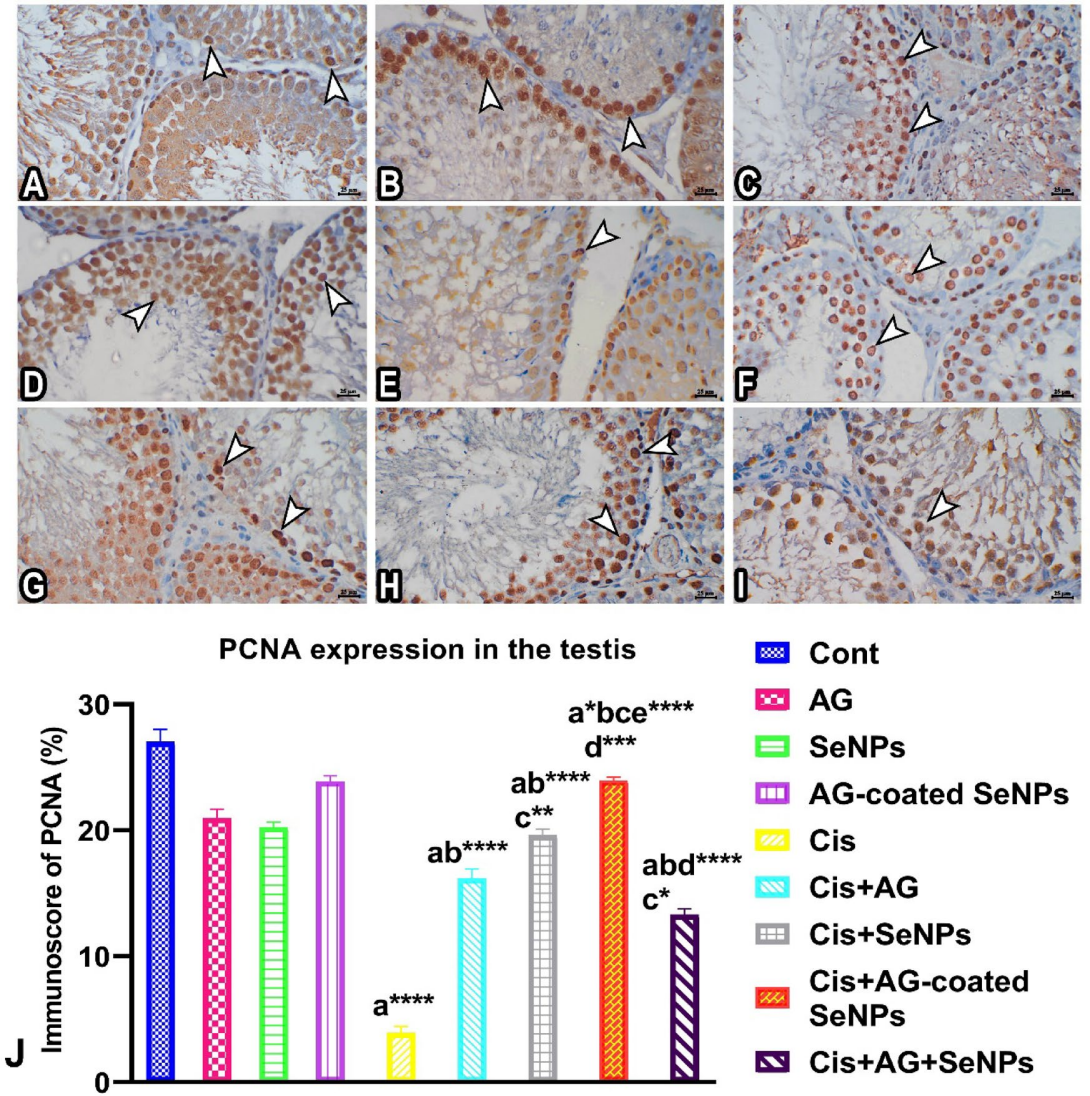
(**A**)–(**J**) Photomicrographs of PCNA immunostained testicular sections (Scale bar A-F = 25 µm). (**A**) Control group, (**B**) AG group, (**C**) SeNPs group, and (**D**) AG-coated SeNPs group showing the uppermost expression of PCNA, (**E**) Cis group showing the lowest expression of PCNA protein, (**F**) Cis + AG group, (**G**) Cis + SeNPs group, showing restoration of PCNA immune-expression intensity, (**H**) Cis + AG-coated SeNPs group showing very high expression of PCNA protein, and (**I**) Cis + AG + SeNPs group depicting a mild restoration if PCNA expression Positive expression of PCNA (white arrowhead). (**J**) Image analysis of PCNA immuno-reactivity (%) in the testes of the control and other experimental groups. Data are expressed as mean ± SE (n = 5 per group). 'a' denotes significance vs. control; 'b' denotes significance vs. Cis, 'c' denotes significance vs. Cis + AG, 'd' denotes significance vs. Cis + SeNPs. 'e' denotes significance vs. Cis + AG + SeNPs, respectively. * (*P* < 0.05), ** (*P* < 0.01), ), *** (*P* < 0.001), and **** (*P* < 0.0001).

Figure 10 illustrates the immunohistochemistry expression of proliferating cell nuclear antigen (PCNA) in testicular tissues across the control and various experimental groups. The control (Fig. 10A), AG (Fig. 10B), SeNPs (Fig. 10C), and AG-coated SeNPs (Fig. 10D) groups exhibited enhanced PCNA expression, indicating active cellular proliferation.

However, PCNA expression in the Cis group (Fig. 10E) was significantly (*P* < 0.0001) reduced compared to the control, indicating that testicular cell proliferation is impaired. On the other hand, the combinations of Cis + AG (Fig. 10F), Cis + SeNPs (Fig. 10G), Cis + AG-coated SeNPs (Fig. 10H), and Cis + AG + SeNPs (Fig. 10I) markedly enhanced PCNA expression relative to the Cis group (*P* < 0.0001), indicating a considerable enhancement in proliferative activity. Additionally, Cis + AG-coated SeNPs (Fig. 10H) exhibited the greatest expression of PCNA across the treatment groups. Furthermore, Cis + AG + SeNPs (Fig. 10I) demonstrated considerable PCNA expression, exceeding that of Cis alone, although remaining below the levels seen in Cis + AG-coated SeNPs.

Figure 10J depicts the assessment of PCNA immunoreactivity percentages, demonstrating that AG, SeNPs, and AG-coated SeNPs alleviated Cis-induced inhibition of cell proliferation, with AG-coated SeNPs showing the most pronounced protective effect.

### Flow cytometric analysis of cytochrome C

**Fig. 11:**
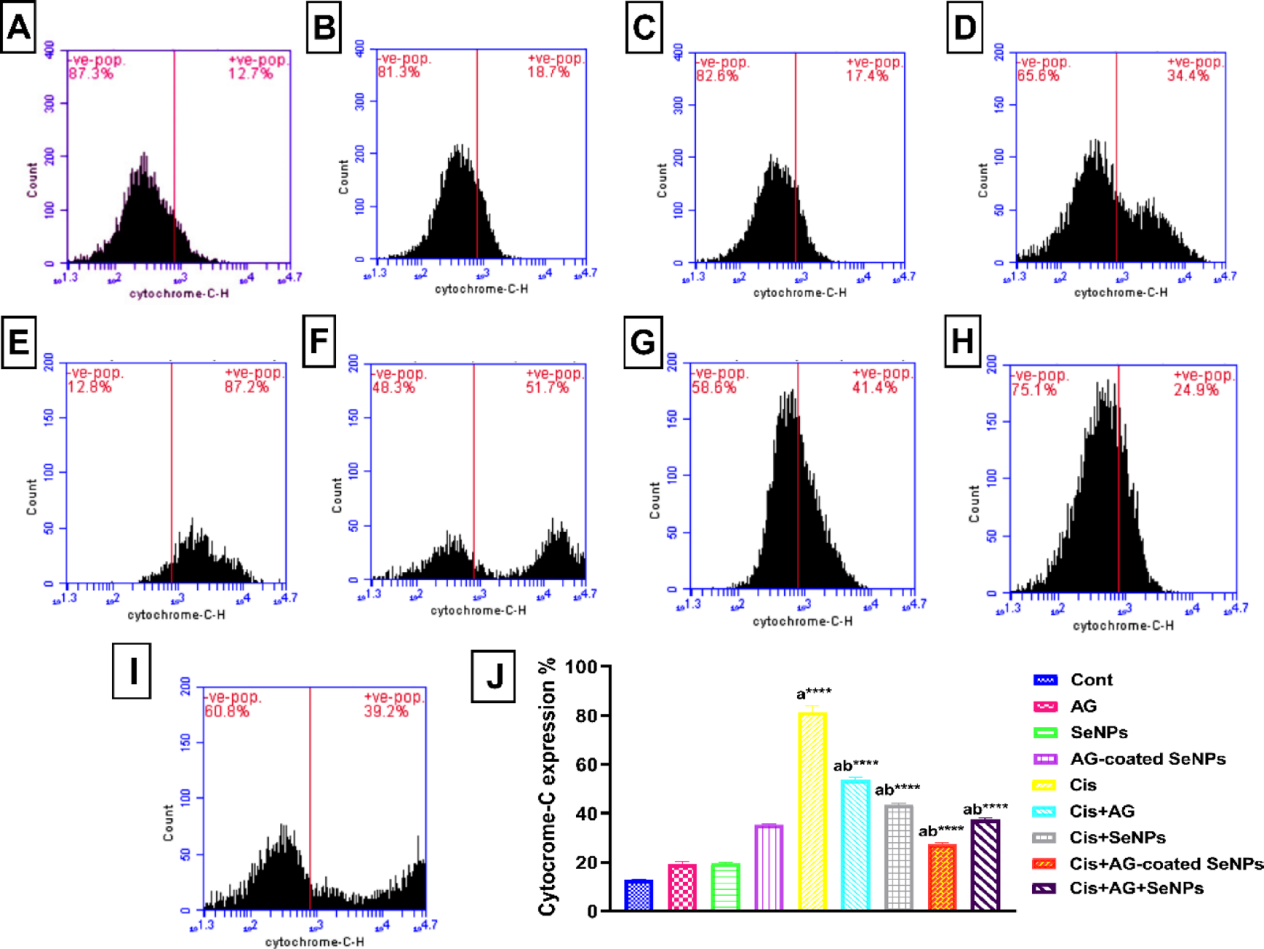
(**A**)–(**J**) Flow cytometric analysis of cytochrome C of (**A**) Control group (12.7%), (**B**) AG group (18.7%), (**C**) SeNPs group (19.4%), (**D**) AG-coated SeNPs group (35.4%), (**E**) Cis group (81.5%), (**F**) Cis + AG group (54.5%), (**G**) Cis + SeNPs (43.4%), (**H**) Cis + AG-coated SeNPs group (27.4%), and (**I**) Cis + AG + SeNPs group (36.9%). (**J**) The histogram of cytochrome C release expression percentage (%) in the testis of the control and other experimental groups. Data are expressed as mean ± SE (n = 5 per group).'a' denotes significance vs. control; 'b' denotes significance vs. cisplatin, respectively. ****(*P* < 0.0001).

The presented flow cytometric Fig. 11: (A–J) illustrates cytochrome C release expression percent across the control and other experimental groups, indicating mitochondrial apoptosis involvement. Control (Fig. 11A), AG (Fig. 11B), SeNPs (Fig. 11C), and AG-coated SeNPs (Fig. 11D) groups exhibited cytochrome C positive cells; 12.7%, 18.7%, 19.4%, and 35.4%, respectively, confirming baseline apoptotic activity. While Cis group (Fig. 11E) exhibited significant (*P* < 0.0001) large proportion of cytochrome C release (81.5%) relative to the control group and this indicates its robust pro-apoptotic activity.

On the other side, the percent of cytochrome C released was significantly (*P* < 0.0001 vs. Cis) lowered, resulting in a partial protective effect of Cis + AG (Fig. 11F) and Cis + SeNPs (Fig. 11G) treatments to 54.5% and 43.4%, respectively. In addition, Cis + AG-coated SeNPs (Fig. 11H) further reduced significantly (*P* < 0.0001 vs. Cis) cytochrome C positive cells to 27.4% indicating improved mitochondrial protection. Furthermore, Cis + Se + AG group (Fig. 11I) demonstrated 36.9% cytochrome C release, which reinforced protective effect of combined antioxidants that showed significant reduction in comparison with Cis group (*P* < 0.0001).

The expression percentage of cytochrome C release (%) of control and other experimental groups is measured in Fig. 11J. These results emphasize that AG-coated SeNPs are very effective in rescuing the Cis-induced apoptosis, probably permitting the mitochondrial stabilization.

### Detection of DNA damage by comet assay

**Fig. 12:**
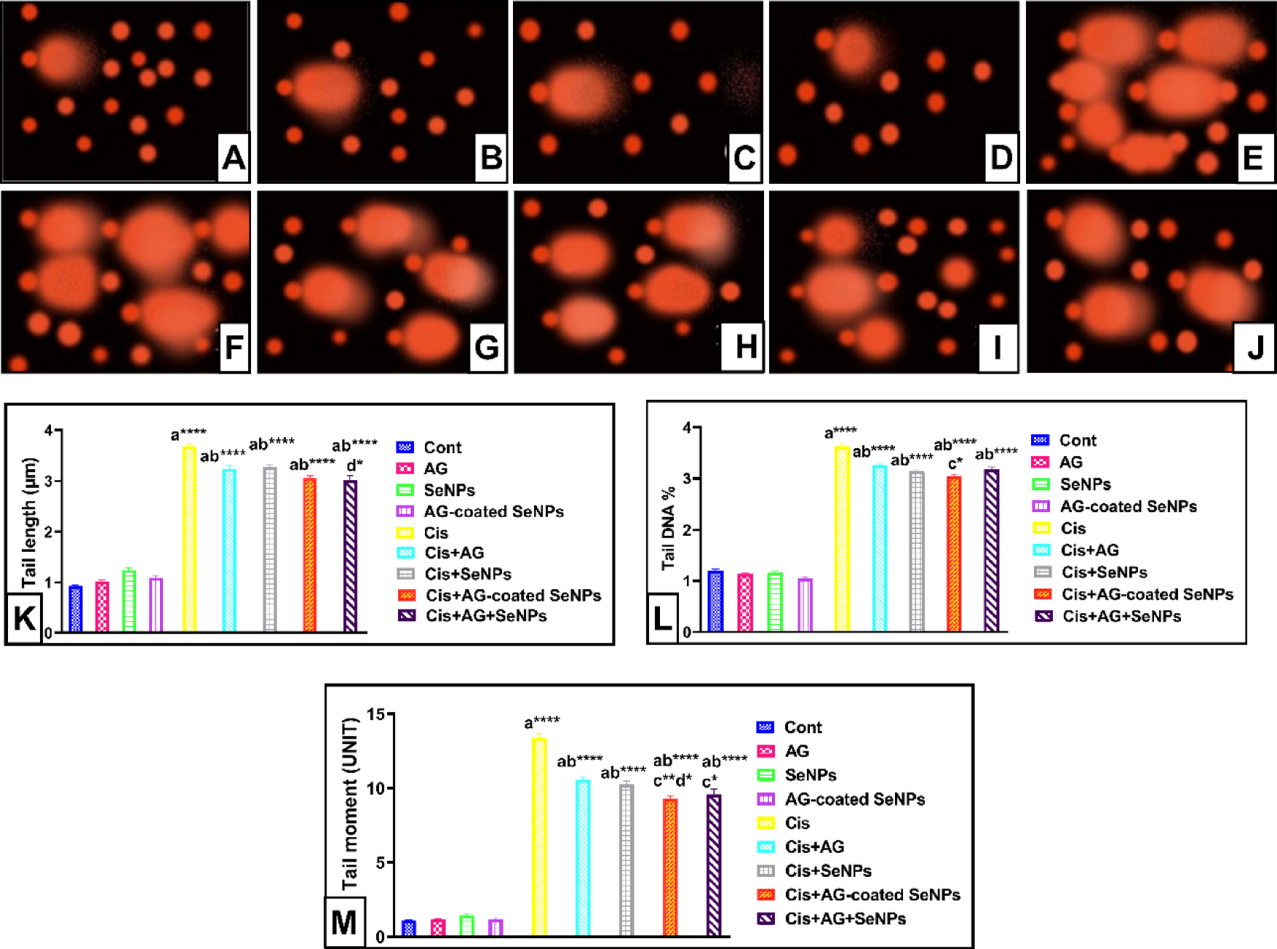
(**A**)–(**M**) Comet assay analysis of (**A**) Control group, (**B**) AG group, (**C**) SeNPs group, (**D**) AG-coated SeNP group, (**E**) and (**F**) Cis group, (**G**) Cis + AG group, (**H**) Cis + SeNPs, (**I**) Cis + AG-coated SeNPs group, and (**J**) Cis + AG + SeNPs group. (**K**) Tail length (µm), (**L**) Tail DNA % and (**M**) Tail moment (UNIT) histograms of the control and other experimental groups. Data are expressed as mean ± SE (n = 5 per group). 'a' denotes significance vs. control; 'b' denotes significance vs. Cis, 'c' denotes significance vs. Cis + AG, 'd' denotes significance vs. Cis + SeNPs. 'e' denotes significance vs. Cis + AG + SeNPs, respectively. * (*P* < 0.05), ** (*P* < 0.01), *** (*P* < 0.001), and **** (*P* < 0.0001).

The results of the comet assay (Fig. 12A–M) indicate that Control (Fig. 12A), AG (Fig. 12B), SeNPs (Fig. 12C), and AG-coated SeNPs (Fig. 12D) groups had a little evidence of DNA damage, short comet tails, and low tail DNA percentage, which suggested no prominent genotoxicity. The quantitative analysis results (Fig. 12K–M) confirmed that tail length (Fig. 12K), tail DNA percentage (Fig. 12L), and tail moment (Fig. 12M) did not exhibit significant differences among these groups.

Contrastingly, all parameters of DNA damage significantly (*P* < 0.0001) increased in the Cis group (Fig. 12E, F) as compared to the control group, which substantiated its genotoxic action. While, AG (Fig. 12G), SeNPs (Fig. 12H), AG-coated SeNPs (Fig. 12I), or AG + SeNPs (Fig. 12J) co-treatments showed a considerable reduction in the markers of DNA damage in comparison with the Cis group (*P* < 0.0001), while, the AG-coated SeNPs group had the strongest protective effect. The quantitative analysis results: (Fig. 12K–M), draw attention to the increase of the geno-protective effects of AG-coated SeNPs on Cis-induced genotoxicity, probably because of enhanced antioxidant activity and cellular delivery.

## Discussion

Infertility is a major global health concern, with male factors contributing to over fifty percent of instances. The administration of chemotherapeutic drugs, particularly cisplatin (Cis), has been shown to adversely affect male fertility via the excessive generation of reactive oxygen species (ROS), resulting in oxidative stress, mitochondrial dysfunction, DNA damage, apoptosis, and compromised semen quality^19^. Disproportion of ROS and antioxidant defenses is explained as one of the primary mechanisms of testicular toxicity caused by Cis^20^.

Natural compounds with antioxidant and anti-inflammatory properties have been investigated to mitigate reproductive damage induced by chemotherapy. Arab gum (AG) is an agent that has been demonstrated to possess antioxidant, anti-inflammatory, and cytoprotective effects^12^. Interestingly, the properties of nanoparticles are unique, including significant specific surface area, high surface activity, numerous surface-active centers, high catalytic and adsorption capacity, as well as low toxicity^21^. As an essential trace element, selenium (Se) is an important element in antioxidant defense systems. Also, it is required in human beings and animals in terms of fertility, growth, and immunity^22^. In addition, selenium nanoparticles (SeNPs) are increasingly gaining attention because they are highly bioactive, less toxic, and possess great bioavailability in comparison with the bulk of selenium^23,24^. The physical characterization of the synthesized AG-SeNPs revealed an apparent size variance between Dynamic Light Scattering (DLS) measurements (peak at 3.39 nm) and Transmission Electron Microscopy (TEM) imaging (58–73 nm). This discrepancy is a well-documented phenomenon in bionanotechnology, stemming from the distinct physical states required by each modality. DLS quantifies the hydrodynamic diameter, which accounts for the primary nanoparticle and its surrounding solvation shell and capping layer in an aqueous, highly dispersed state^25^. In this medium, the Acacia senegal (Arab Gum) coating acts as a potent electrosteric stabilizer, preventing inter-particle van der Waals attraction and maintaining the ultra-small primary particle size.

Conversely, TEM analysis involves solvent evaporation and dehydration on a copper grid. This process collapses the hydration layer of the AG matrix and subjects the particles to intense capillary forces, driving the ultra-small primary units to assemble into larger secondary agglomerates^26^. Consequently, while TEM provides insight into the morphology and clustering behavior of the formulation in a solid state, the DLS data more accurately represents the highly dispersed, ultra-small state of the nanoparticles as they would exist within physiological fluids and cellular environments. The chemical composition of the capping agent was further elucidated via GC–MS. While *Acacia senegal* is primarily recognized as a complex polysaccharide-glycoprotein matrix, our analysis predominantly identified a phytochemical fraction consisting of terpenes and fatty acids. This is consistent with the selective nature of standard GC–MS methodology, which detects volatile and semi-volatile organic compounds; the massive, non-volatile polysaccharide backbone of AG remains undetectable without specialized derivatization^27^. Thus, the identified compounds represent the bioactive “fingerprint” of the plant exudate.

Mechanistically, the AG-capping offers a synergistic dual advantage in the treatment of Cisplatin (Cis)-induced gonadotoxicity. The primary polysaccharide matrix ensures structural stability and enhances the bioavailability required for targeted delivery to the testicular tissue^28^. Simultaneously, the minor phytochemical constituents specifically the identified terpenes and unsaturated fatty acids are well-known for their inherent antioxidant and anti-inflammatory properties^29^. These secondary metabolites likely act in concert with the Selenium core to quench reactive oxygen species (ROS) and suppress the lipid peroxidation cascade triggered by Cisplatin. This multifaceted defense mechanism preserves the integrity of the seminiferous tubules and maintains the spermatogenic capacity, as evidenced by the restored sperm count and motility observed in the AG-SeNP treated cohorts.

The findings of the current study demonstrated that SeNPs, AG, and AG-coated SeNPs played protective effects against Cis-induced toxicity via a series of biochemical and molecular detective methods. The results revealed significant changes in body weight, oxidative stress, inflammatory and fibrotic responses, activity of apoptotic cell, and genotoxic effects. These remarkable changes in the body weight and seminal vesicles relative weight after Cis treatment are consistent with the previously observed evidence that suggests such changes are caused by the systemic cytotoxic activities of Cis, resulting in weight loss and multi-system tissue damage, including harmful effects on testes^30^.

Conversely, the co-administration of SeNPs, AG, and especially AG-coated SeNPs markedly restored body weight. This protective effect aligns with prior research indicating that AG mitigates the negative effects of anticancer drugs such as Cis, particularly via its potent antioxidant, antibacterial, antiviral, and anti-inflammatory properties^12^. Furthermore, substances containing selenium have been documented in a prior research to mitigate toxicity generated by Cis, perhaps owing to their antioxidant capabilities^31^, demonstrated that SeNPs possess protective characteristics for body and organ weights following the administration of Cis.

Surprisingly, despite the significant recovery of body weight and relative seminal vesicles weight in the co-treated groups, the testes and epididymides weights did not change considerably. The given finding is opposed by the results of Oda et al., 2025^32^, who have revealed higher increase in the weights of reproductive organs after selenium supplementation. This could be as a result of differences in dosage, length of treatment, or in the design of the experiment. Nevertheless, the AG-coated SeNPs group reported the greatest increase because of a stabilizing effect of AG that increases the bioavailability of SeNPs^33^.

The administration of Cis resulted in a significant decline in catalase (CAT) and glutathione peroxidase (GPX) activity, with a substantial elevation in malondialdehyde (MDA) levels, so confirming the onset of oxidative stress. These findings align with those shown by^34^, who demonstrated that Cis promotes oxidative stress, resulting in cellular damage. Treatment with AG, SeNPs, and particularly AG-coated SeNPs, effectively restored CAT and GPx activity while reducing MDA levels, demonstrating their significant antioxidant potential. This finding aligns with our recent research demonstrating that SeNPs enhance antioxidant defenses against oxidative damage induced by Cis^31^. The fact that AG-coated SeNPs was found to have superior protective effects in the current study can also be referred to prior research, which implies that the coating of SeNPs with antioxidant agents like AG can improve the bioavailability of SeNPs and synergize their antioxidant effects^33^.

The upregulation of transforming growth factor beta (TGF-β) and interleukein-6 (IL-6) in the Cis group is evidence that fibrotic and inflammatory signal transduction is stimulated, which is in line with the results of previous studies^35^. On the other hand, the significant reductions seen in these cytokines following the co-administration of AG, SeNPs, and AG-coated SeNPs indicated the ability of these agents to inhibit inflammation and fibrosis complementing previous studies that reported a role of selenium and antioxidant agents in stabilizing inflammatory responses^36^.

Interestingly, the suppressive properties of AG-coated SeNPs on TGF-β and IL-6 were most pronounced, which is expected to be explained by the ability of AG to stabilize SeNPs, thus contributing to increased protection capacity and bioavailability^37^. The results of fibrotic indicators were corroborated by Masson’s trichrome staining, which was used to assess collagen fiber deposition in the testicular tissue. The Cis group had a substantial accumulation of blue-stained collagen fibers, particularly in the testicular capsule. Additional investigations have similarly shown heightened collagen deposition in the tunica albuginea and arterial walls^38^. This increase may be attributed to the activation of p38 mitogen-activated protein kinase by reactive oxygen species, which then initiates TGF-b1-mediated fibrotic processes^39^. Conversely, the co-treatment groups had a substantial reduction in these fibers, whilst the Cis + AG-coated SeNPs group exhibited a considerable decrease, which demonstrates a powerful anti-fibrotic response. This outcome aligns with previousstudy^40^.

The potent anti-inflammatory response observed via flow cytometry, characterized by a marked reduction in TNF-α expression, directly correlates with the unique chemical and structural profile of the AG-SeNP formulation. TNF-α is a master regulator of the inflammatory cascade; its overproduction often leads to downstream tissue damage and apoptosis. The significant reduction of this cytokine in the AG-coated SeNP group suggests that the coating not only improves the stability of the selenium core but also enhances its ability to interface with immune signaling pathways.

While Cisplatin-induced systemic toxicity is mediated by a cytokine storm and the up-regulation of pro-inflammatory markers, the AG-coated SeNPs effectively dampened this response. This efficacy is likely a direct result of the synergy between the elemental Selenium core and the bioactive phytochemicals specifically terpenes and unsaturated fatty acids identified in our GC–MS analysis. Terpenes have been extensively documented to exert anti-inflammatory effects by inhibiting the NF-κB signaling pathway, thereby reducing the production of pro-inflammatory cytokines like TNF-α^41^. Furthermore, the superior performance of the nano-conjugate compared to the physical mixture (Cis + AG + SeNPs) underscores the importance of the nano-formulation in achieving optimal bioavailability. By stabilizing the Selenium core within the AG matrix, the formulation ensures the concurrent delivery of antioxidant selenium and anti-inflammatory secondary metabolites to the cellular environment. This localized delivery likely quenches the oxidative triggers of inflammation more efficiently than the individual components, thereby protecting the testicular tissue from secondary inflammatory damage and preserving the overall integrity of the reproductive system^42^.

Histopathological analysis utilizing H&E staining provided additional confirmation of substantial testicular damage in the Cis group, characterized by distortion of normal testicular architecture, notable widening of interstitial spaces surrounding seminiferous tubules, and pronounced vascular congestion. These findings are consistent with prior research^38^. Vascular congestion is considered an indicator of Cis-induced inflammation, linked to elevated activation of nuclear factor kappa B. The enlarged interstitial spaces suggest interstitial edema, likely resulting from Cis-induced endothelial damage that initiates telangiectasia, followed by endothelial injury and fluid leakage.

Moreover, Cis has also been reported to induce excessive nitric oxide synthesis that causes vasodilation and hypotension, which eventually results in a decrease in organ perfusion and edema^40^. The seminiferous tubules in this group were narrow and irregular, with a convoluted basement membrane, with either partial or complete degeneration of the germinal epithelium. Pyknotic nuclei were seen in Leydig cells. These findings align with previous research^43^. The superior efficacy of the AG-coated nano-formulation likely stems from a synergistic interaction between the antioxidant properties of Selenium and the stabilizing, biocompatible nature of Arabic Gum. By enhancing the bioavailability of SeNPs, the AG coating may facilitate a more efficient neutralization of Cis-induced reactive oxygen species (ROS) within the testicular tissue. This protection appears critical in maintaining mitochondrial function (as evidenced by restored motility) and preventing DNA damage that leads to morphological defects. The marked difference between the coated nanoparticles and the physical mixture underscores the importance of the nano-conjugation process in achieving optimal therapeutic indices for preventing chemotherapeutic-induced infertility.

Conversely, the administration of AG, SeNPs, and AG-coated SeNPs led to observable improvements in testicular histology, with the highest level of tissue recovery in AG-coated SeNPs. These findings match previous researchers that suggest that selenium-related therapies improve tissue healing and provide defense against Cis-induced testicular injury^12^.

Caspase-3, an indicator of apoptotic activity, exhibited a significant elevation in the Cis group, corroborating the pro-apoptotic impact of Cis. Nonetheless, its expression was negligible in the groups co-treated with AG, SeNPs, and AG-coated SeNPs, therefore affirming their anti-apoptotic effects. This observation aligns with the findings of^40^, which demonstrated that selenium compounds may mitigate apoptosis in diverse organs.

Prior studies have shown that SeNPs mitigate oxidative stress by obstructing caspase-dependent apoptosis triggered by Cis, mostly by reducing the overproduction of ROS^44^ and by elevating the levels of the antioxidant metallothionein-1 in testicular tissue^45^. The AG-coated SeNPs group exhibited the most pronounced decrease in Caspase-3 expression, highlighting their superior cytoprotective efficacy, attributed to AG’s capability to stabilize SeNPs and enhance their bioavailability^37^. Interestingly, the elevated baseline of Cytochrome C observed in the AG-SeNP control group did not correlate with Caspase-3 activation or histological damage. In the unique physiological context of the testis, this molecular profile likely signifies enhanced mitochondrial biogenesis and oxidative phosphorylation capacity^46,47^. Given the significant energetic requirements of spermiogenesis, this AG-SeNP-induced mitochondrial ‘priming’ appears to support the observed increase in spermatid maturation and structural integrity, highlighting the dual role of both Selenium and Arab gum specially on their nano formulation as both a metabolic enhancer and a structural component of the sperm mitochondrial sheath^48,49^.

Immunohistochemical examination of proliferating cell nuclear antigen (PCNA) revealed a significant reduction of PCNA in Cis group, attributable to Cis-induced toxicity leading to impaired cell proliferation in the testicles. This finding is consistent with other studies indicating that Cis disrupts spermatogenesis by generating oxidative stress and DNA damage^50^. Conversely, the co-administration of AG, SeNPs, and particularly AG-coated SeNPs, resulted in a notable restoration of PCNA expression, indicating an enhanced proliferative capacity. The AG-coated SeNPs group exhibited the most pronounced enhancement, nearing the levels of the control group, likely attributable to the synergistic effects of AG and selenium’s antioxidant and protective characteristics, together with improved cellular absorption^51^.

The comet test revealed considerable DNA damage in the Cis group, but co-treatment with AG, SeNPs, and AG-coated SeNPs notably reduced DNA damage, particularly in the AG-coated SeNPs group. This supports the concept that AG-coated SeNPs have superior DNA-protective properties, likely due to their enhanced antioxidant and anti-apoptotic actions. The result aligns with previous research indicating selenium’s efficacy in preventing genotoxicity induced by Cis^52^. Finally, It is important to note the translational context of the experimental model utilized in this study. Clinical Cisplatin therapy typically involves cumulative, multi-cycle dosing, whereas the current study employed a single acute intraperitoneal injection (5 mg/kg). This acute model is a widely validated approach designed to induce severe, reproducible oxidative stress and germ cell depletion while minimizing systemic lethality in rodents. Furthermore, the 45-day post-insult treatment duration was specifically tailored to span the approximate length of one complete spermatogenic cycle in Wistar rats (~ 48 to 52 days). This allowed for a comprehensive evaluation of AG-SeNP-mediated protection across all stages of germ cell development and maturation. While this acute injury model provides crucial mechanistic proof-of-concept, future studies employing chronic, fractionated Cisplatin dosing models will be beneficial to further validate the long-term clinical applicability of AG-SeNPs in preventing cumulative chemotherapy-induced infertility^53–55^.

The data suggest that a direct coating of SeNPs with AG enhances their efficacy in mitigating Cis-induced testicular damage. This protection is likely superior due to enhanced bioavailability and synergistic antioxidant effects. Consequently, AG-coated SeNPs have a more pronounced protective effect against Cis-induced testicular injury compared to the administration of AG and SeNPs individually or as a simple combination.

## Conclusions

This study demonstrates that the physical coating of Selenium Nanoparticles with Arab Gum (AG-SeNPs) offer superior protection against Cisplatin-induced testicular toxicity compared to uncoated SeNPs or a mere physical mixture. The primary novelty lies in the synergistic dual action of the AG coating, which simultaneously ensures structural stability for enhanced bioavailability and provides intrinsic antioxidant properties to alleviate localized tissue damage. While AG-SeNPs show considerable promise as a fertility-preserving adjunctive therapy during platinum-based chemotherapy, advancing this formulation toward clinical application requires specific follow-up. Future research must prioritize targeted in vitro mechanistic studies to delineate precise intracellular pathways, comprehensive pharmacokinetic profiling to track in vivo biodistribution and clearance, and rigorous dose optimization to establish long-term reproductive safety and the ideal therapeutic window."

## Data Availability

The datasets generated and analyzed during the current study are ‎available from the corresponding author on reasonable request.‎

## Abbreviations

Cis: Cisplatin
DNA: Dioxyribonucleic acid
SeNPs: Selenium nanoparticles
AG: Arab gum
AG-coated SeNPs: Arab gum-coated selenium nanoparticles
WHO: World Health Organization
ROS: Reactive oxygen species
TEM: Transmission electron microscopy
UV: Ultraviolet
FT-IR: Fourier transform infrared
XRD: X-ray diffraction
GC–MS: Gas Chromatography–Mass Spectrometry
VACSERA: The Holding Company for Biological Products and Vaccines
CAT: Catalase
GPx: Glutathione peroxidase
MDA: Malondialdehyde MDA
TGF-β: Transforming growth factor beta
IL-6: Interleukin-6
ELISA: Enzyme-Linked Immunosorbent Assay
PCNA: Proliferating cell nuclear antigen
DLS: Dynamic Light Scattering
PDI: Polydispersity index
RT: Retention time
H&E: Hematoxylin and Eosin
